# Solar-Assisted Membrane Distillation

**DOI:** 10.3390/membranes12030304

**Published:** 2022-03-09

**Authors:** Chii-Dong Ho

**Affiliations:** Department of Chemical and Materials Engineering, Tamkang University, Tamsui, New Taipei 251, Taiwan; cdho@mail.tku.edu.tw

The integration of solar power and solar thermal systems using sunlight as the fuel can work in remote arid areas to meet the freshwater demand with membrane desalination processes, which is important in considering both the low environmental impact and the production cost. Solar energy is the most promising substitute for fossil fuels, which affect the regional and global environment. The use of solar energy offers an alternative selection to decrease the dependence on fossil fuels and production of harmful emissions as well. Solar energy can be converted into electricity and thermal heat using photovoltaics (PV) and solar collectors, respectively. The combination of solar thermal and PV energy (or thermal/PV hybrid) with membrane desalination (MD) has proven to be technically feasible and widely recognized in saline water purification. Different MD processes using thermal-based and pressure-driven methods have been implemented with solar energy resources for corrosion-free heat exchangers. The membrane distillation (MD) process is non-isothermal to derive permeate flux across a hydrophobic porous membrane from high to low vapor pressure with a hybrid of thermal distillation and membrane processes. Advantageous conditions of lower operating temperatures in phase-change desalination systems generate the transmembrane evaporation processes which result in a vapor pressure gradient across a hydrophobic porous membrane. The feed saline water can be heated in membrane distillation processes by solar-thermal devices given the moderate operating temperatures (50–80 °C). In recent years, relevant studies have worked practicably on coupling membrane desalination systems with solar energy technologies in many pilot and commercialized plants. Technological assessments such as small or large equipment units, energy consumption, and fouling problems have been examined in the nexus of technical feasibility and economic benefits with the aim of creating integrated systems of a solar-assisted thermal-driven membrane distillation system. The overall objective of the solar-assisted membrane desalination system was its application in arid and semi-arid remote regions, where there is brackish or abundant natural seawater along with high solar irradiation to produce high purity water for domestic use. In addition, solar-assisted membrane seawater desalination processes have become economical and technically feasible for water drinkability due to the improvement of membrane materials and technologies.

This Special Issue titled “Solar-Assisted Membrane Distillation” in the journal Membranes aims to examine the performance of devices such as solar-driven membrane distillation (SDMD) modules under various operating conditions and membrane parameters including air gap membrane distillation (AGCMD), direct contact membrane distillation (DCMD), permeate gap membrane distillation (PGMD), and vacuum membrane distillation (VMD). There are a total of four research articles that contribute to this Special Issue.

Chen et al. [1] presented a two-stage design approach for the SDMD systems using different types of membrane distillation configurations, including AGMD (air gap MD), DCMD (direct contract MD), and VMD (vacuum MD) by employing the steady state and dynamic simulation models with the platform of the Aspen Custom Modeler^®^ (ACM) simulator. Two design stages determine equipment sizes under the minimized total annual cost (TAC) scenario as well as the SDMD systems with process control to adjust the operating flow rates. As an illustration, the unit production costs (UPCs) of the optimal SDMD systems using AGMD, DCMD, and VMD were operated with the yearly solar radiation intensity of Taiwan using the dynamic model. Both TAC and UPC of the solar-driven AGMD system are the lowest among various SDMD systems, which can be reduced from US$ 2.71/m^3^ to US$ 2.04/m^3^, while the membrane unit cost decreases from US$ 90/m^2^ to US$ 36/m^2^.

Chang et al. [2] investigated the promotion of both the mass and heat transfer characteristics to improve the productivity of pure water from direct contact membrane distillation (DCMD) modules at the middle operation temperature (45 °C to 60 °C) of hot inlet saline water. The permeate flux processed by inserting the manufacturing 3D turbulence promoter into DCMD modules was investigated theoretically and experimentally with hot inlet saline temperatures and flow rates set as parameters. Two geometric shapes of three-dimensional (3D) printing cubiforms (circular and square of same surface areas), treated as eddy promoters of different array patterns, were inserted into the flow channel of the DCMD device to create vortices in the flow stream and to increase turbulent intensity. The additive manufacturing 3D turbulence promoters to promote both the mass and heat transfer characteristics could not only prevent the membrane from vibration but also augment the permeate flux via lessening the thermal-boundary layer. The decreased coefficients of the temperature polarization were achieved under various geometric shapes and both concurrent- and countercurrent-flow patterns. Thus, the permeate flux enhancement for the DCMD module in this study, by inserting 3D turbulence promoters, could provide a maximum relative increment of up to 61.7%, compared to those in the empty channel device. An optimal design of the DCMD module with the insertion of 3D turbulence promoters was also evaluated in terms of the ratio between permeate flux enhancement and power consumption increment.

Alquraish et al. [3] reported a new prototype of a membrane distillation plant with sunlight as the only source of energy for water desalination under the climate condition of Kairouan City, Tunisia in August 2020. The desalination plant consists of solar energy collectors and photovoltaic panels for heating brackish water as well as a compact arrangement of the spiral wound membrane design allowing for effective internal heat recovery. The trial investigation of the pilot solar-powered saturate hole membrane distillation was based on the observation from Gained Output Ratio (GOR) of the layer module that relies upon the day-by-day sunlight-based radiation. The GOR after-effects of this framework were in the scope of 0.3–1.4.

Shuldes et al. [4] reviewed an energy efficiency analysis for a solar-thermal desalination scheme of a hollow fiber vacuum membrane distillation (VMD) module using finite element analysis under various operating conditions and membrane parameters. The polarization of salt concentration within the boundary layer rapidly increased with the limiting phenomenon where the performance of permeate flux under VMD operation was inhibited. Adding a baffling scheme to the surface of the fibers can break the mass-transfer boundary layer and effectively mitigate the polarization effect, therefore improving the water recovery. Increasing the overall recovery ratio was found to enhance energy efficiency, especially when using multiple stages of VMD modules, which were nearly not dependent on membrane characteristics from an energy standpoint. Furthermore, the results show that improvements in solar energy collection and process designs allow energy to be reused more effectively.

In conclusion, the novel findings from these studies reveal the importance of solar-assisted membrane distillation for desalination processes. Numerous design schemes have proven the considerable improvements in various membrane distillation modules. Additionally, those results are promising for further in-depth inquiry and could be suitable for practical applications. This Special Issue introduces guidelines and the potential practicability for the development of these desalination processes in a sustainable way.

## Data Availability

Not applicable.
